# Developing highER-throughput zebrafish screens for *in-vivo* CNS drug discovery

**DOI:** 10.3389/fnbeh.2015.00014

**Published:** 2015-02-12

**Authors:** Adam Michael Stewart, Robert Gerlai, Allan V. Kalueff

**Affiliations:** ^1^ZENEREI Institute and The International Zebrafish Neuroscience Research ConsortiumSlidell, LA, USA; ^2^Department of Psychology, University of Toronto MississaugaON, Canada; ^3^Research Institute for Marine Drugs and Nutrients, College of Food Science and Technology, Guangdong Ocean UniversityZhanjiang, Guangdong, China

**Keywords:** high-throughput screens, zebrafish models, big data, CNS drug discovery, phenomics

## Abstract

The high prevalence of brain disorders and the lack of their efficient treatments necessitate improved *in-vivo* pre-clinical models and tests. The zebrafish (Danio rerio), a vertebrate species with high genetic and physiological homology to humans, is an excellent organism for innovative central nervous system (CNS) drug discovery and small molecule screening. Here, we outline new strategies for developing higher-throughput zebrafish screens to test neuroactive drugs and predict their pharmacological mechanisms. With the growing application of automated 3D phenotyping, machine learning algorithms, movement pattern- and behavior recognition, and multi-animal video-tracking, zebrafish screens are expected to markedly improve CNS drug discovery.

Come on, feel the noise

So jump a little higher,

Jump, jump a little higher…

Scooter, “We Take You Higher” (1996)

## Zebrafish screens for CNS drug discovery

Brain disorders are complex multifaceted illnesses with poorly understood causes and frequently ineffective therapies (Duman et al., 1994; Nestler, 2013). Despite the growing public health impact of these disorders (Mitchell, 2011), the central nervous system (CNS) drugs have not improved in decades (WHO, 2008; Griebel and Holmes, 2013), necessitating novel pre-clinical *in-vivo* models for drug discovery (Markou et al., 2009; Stewart and Kalueff, 2013, 2014).

Research in this field is determined by the complex nature of CNS syndromes, the importance of targeting their biological mechanisms, and the need in high-throughput screens (HTS) for drug targets and potential therapies (Kokel and Peterson, 2008; Rihel et al., 2010; Laggner et al., 2012; Stewart and Kalueff, 2014). With the recent progress of medicinal chemistry, mathematical modeling and bioinformatics, drug discovery has started to embrace systematic, large-scale screening approaches (Bruni et al., 2014). As a vertebrate species with high genetic and physiological homology to humans, the zebrafish (*Danio rerio*) is rapidly emerging as an excellent model to address these needs (Kalueff et al., 2014a,b; Stewart et al., 2014a).

Importantly, using zebrafish as a first-choice vertebrate species for screening has several advantages. First, it helps narrow down the list of potential “hits” for their subsequent validation in more complex and expensive rodent tests. Second, it helps assess drug responses following various genetic manipulations, the toolbox for which is becoming increasingly diverse and efficient in zebrafish (Bernier et al., 2014; Kalueff et al., 2014b; Stewart et al., 2014a). Finally, larval and adult zebrafish screens are also useful for dissecting the drugs' psychopharmacological profiles (e.g., using multiple receptor agonists and antagonists prior to a more targeted rodent testing)—an approach that focuses on “core,” evolutionarily conserved (and, thus, translationally more relevant) molecular pathways shared by zebrafish and humans (Kalueff et al., 2014a,b; Stewart et al., 2014a).

Complementing larval zebrafish HTS, extensively used for modeling brain disorders and testing new compounds, adult zebrafish *in-vivo* testing is often performed as low-to-moderate neurophenotypic screens (Gerlai et al., 2000; Baraban et al., 2005; Kily et al., 2008; Maximino et al., 2010, 2011, 2013, 2014b; Mathur and Guo, 2011; Mathur et al., 2011a,b; Norton et al., 2011; Pan et al., 2011; Rosemberg et al., 2011; Griffiths et al., 2012; Lange et al., 2012a,b; Parker et al., 2013; Ziv et al., 2013; Stewart et al., 2014b; Li et al., 2015).

**Figure 1:**
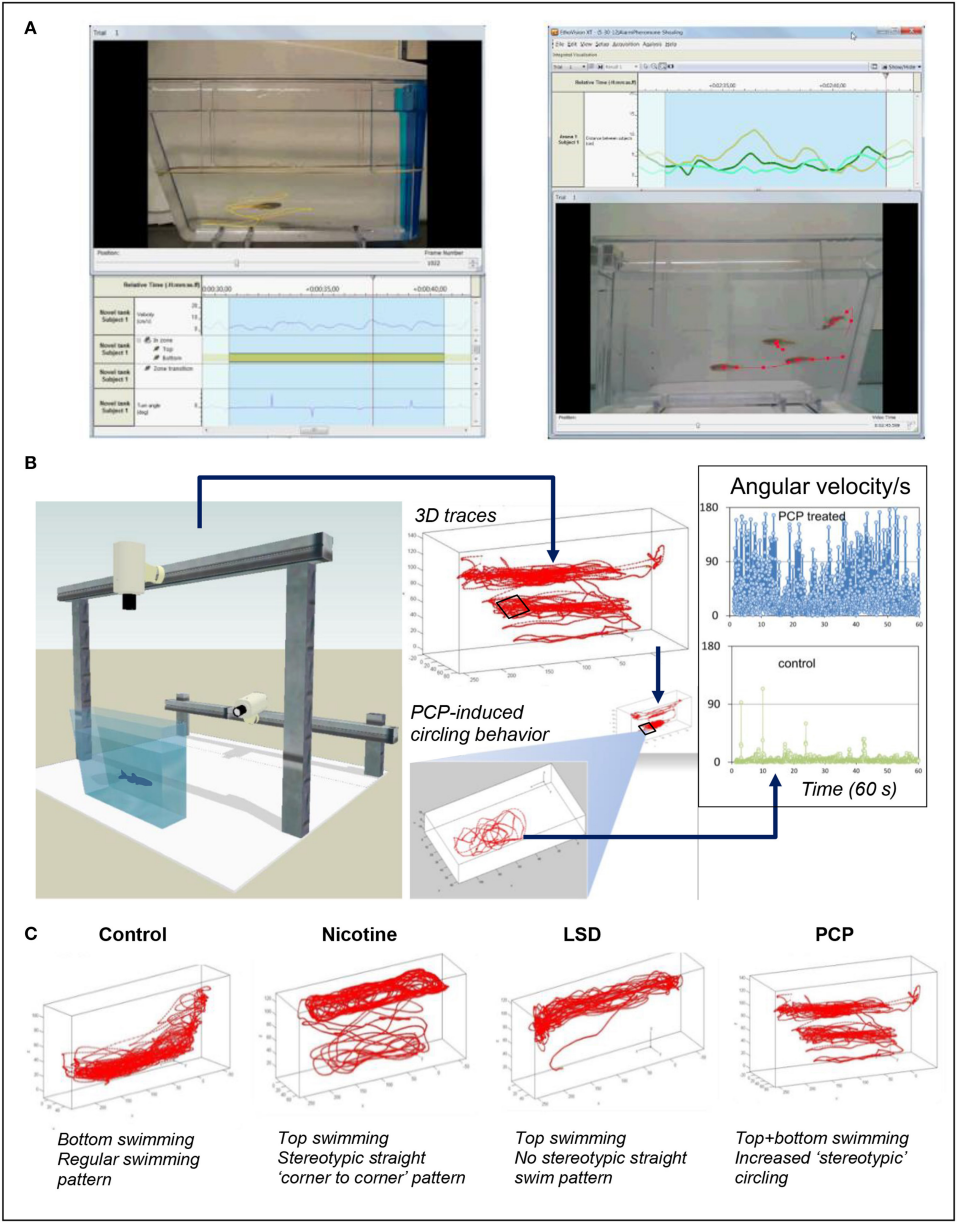
**The use of video-tracking tools to assess neural phenotypes in zebrafish. (A)** Shows video-tracking of an individual zebrafish (left) or a zebrafish group (shoal, right); side view vide-recording in the novel tank test. Tracking individual fish with one camera in 2D, or with two cameras in 3D, can generate up to 50–60 individual endpoints (see Supplementary Table 1S online for examples) which can be sensitive to neuroactive properties of the drugs. Tracing selected endpoints in zebrafish shoals, such as assessing the average inter-fish distance and velocity, is also possible in zebrafish models (Green et al., 2012) (although more sophisticated computer tools and optimized animal tagging methods are needed to monitor each individual fish within the group). **(B)** Illustrates the potential of 3D behavioral video-tracking in zebrafish to predict drug pharmacology (also see Soleymani et al., 2014). In this example, top swimming combined with elevated angular velocity in zebrafish treated with a hallucinogenic drug phencyclidine (PCP, inset) shows a striking difference from control fish, supporting the value of various computer-based neural phenotypes for predicting the pharmacological profile of different CNS-active compounds. (**C)** Shows examples of representative 3D phenotypes for control fish and animals acutely exposed to several CNS drugs. LSD, Lysergic acid diethylamide (images: courtesy of Noldus IT, Netherlands, in collaboration with the Kalueff Laboratory, Stewart et al., 2014a). Note distinct patterns of locomotion evoked by drugs from different pharmacological classes (also see Cachat et al., 2011, 2013; Soleymani et al., 2014 for discussion).

Current *in-vivo* zebrafish HTS typically utilize *extensive* or *intensive* approaches to generate “big data” for translational neuroscience research (Figure 1). For instance, applying a barcoding strategy (Glossary), extensive analyses of several basic locomotor and sleep/wake parameters in larval zebrafish have successfully identified neuroactive drugs from a large library of screened compounds (Kokel and Peterson, 2008, 2011; Kokel et al., 2010; Rihel et al., 2010; Laggner et al., 2012; Jin et al., 2013). Recent *intensive* studies analyzing 20–30 three-dimensional (3D) behavioral endpoints in adult zebrafish (Glossary, Supplementary Table 1S online), have detected potential commonalities and differences in profiles of several tested neuroactive drugs (Egan et al., 2009; Grossman et al., 2010; Wong et al., 2010; Cachat et al., 2011, 2013).

**Figure 2:**
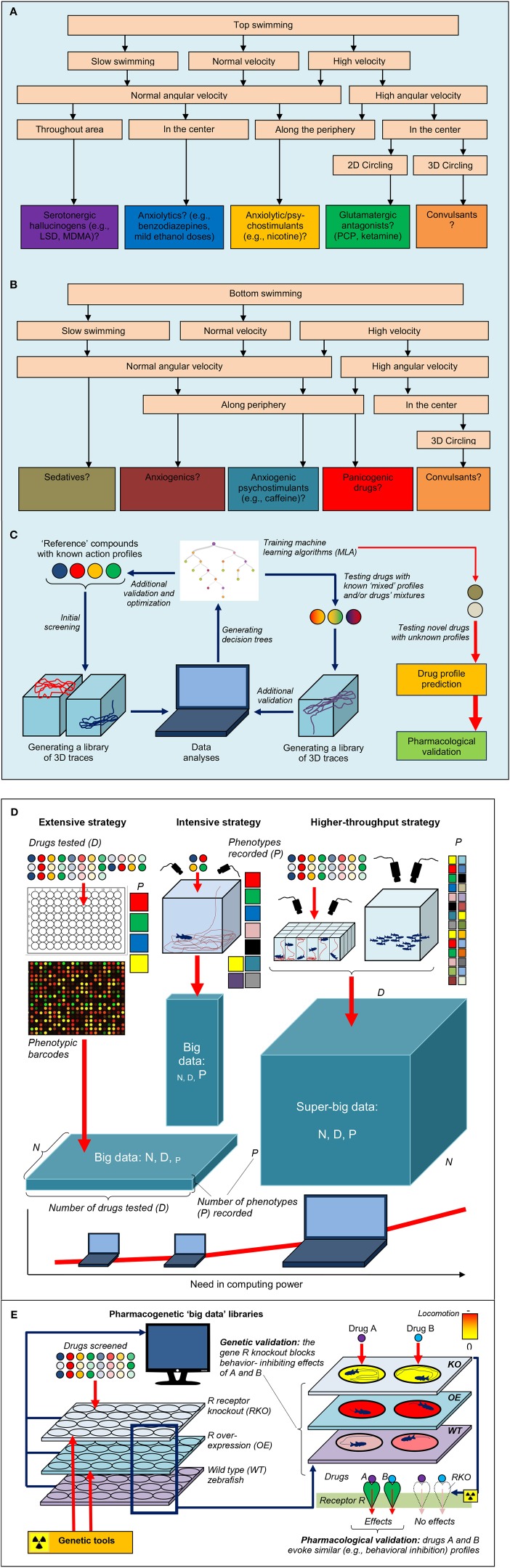
**Example of potential decision trees **(A,B)** that can be used by automated *in-vivo* drug-screening platforms in adult zebrafish to predict the drugs' pharmacological profiles (see Figures 1B,C for examples of 3D traces)**. **(C)** Illustrates the general strategy of drug screening based on machine learning algorithms and 3D trace analyses. Summary of different strategies that can be used to generate high-density biological “big data” from zebrafish *in-vivo* screens. **(D)** Illustrated the extensive approach, testing a large number of drugs (D) in multiple animals (N) but recording few endpoints/phenotypes (P). This approach is markedly facilitated by using phenotypic barcoding approaches (Glossary). In contrast, the intensive approach screens few drugs, uses few animals but records many endpoints. The higher-throughput strategy, based on screening many compounds with multiple endpoints in a large number of animals, is empowered by locomotor pattern and behavioral recognition (Glossary) as well as automated slimuli delivery and experimental manipulations. **(E)** Shows the value of increased drug data “dimensionality” by including pharmacogenetic results (from wild type vs. mutant zebrafish) for providing important mechanistic insights into the drugs action. For example, a hypothetical antagonism of a drug A at a receptor R can be confirmed by screening the reference compound B (with known anti-R activity) and by mutating zebrafish gene *R* to abolish A/B-like activity in the mutants. Applying bioinformatics-based approaches and combining both lines of such evidence will facilitate the discovery of anti-R compounds (based on A-like pharmacology in zebrafish), followed by subsequent target validation in rodents and clinical studies.

Figures 1, 2 further illustrate how a comprehensive evaluation of individual compounds can foster objective, computer-based prediction of the drugs' pharmacology. For example, phencyclidine (PCP) is a hallucinogenic glutamatergic antagonist that acutely evokes characteristic “top circling” behavior in zebrafish (Figures 1, 2). Assessed by elevated angular velocity and rotation index, this response is similar for anti-glutamatergic hallucinogens, but not other classes of hallucinogenic agents (Kyzar et al., 2012; Neelkantan et al., 2013; Stewart et al., 2013). Analyzing such 3D profiles, it is therefore possible to generate decision trees for predicting the pharmacological profile of different groups of neuroactive compounds, based on zebrafish swimming patterns and their geometry (Figure 2) (Cachat et al., 2010, 2011; Soleymani et al., 2014). Likewise, acute nicotine evokes characteristic top swimming along perimeter of the tank, strikingly differing from top circling (typical for many anti-glutamatergic drugs) or top surfacing without peripheral swimming (typical for serotonergic agents; Figure 1C) (Kyzar et al., 2012; Neelkantan et al., 2013; Stewart et al., 2013). Importantly, such accumulation of large libraries of drugs' behavioral signatures in zebrafish can parallel the application of machine learning algorithms, leading to further refinement and optimization of the prediction of drugs' pharmacological profiles (Soleymani et al., 2014) (Figure 2C).

## Developing higher-throughput zebrafish screens

The choice between *extensive* and *intensive* analyses in zebrafish models no longer poses a critical dilemma for researchers, because modern information technology tools and the low cost of zebrafish (vs. rodent) assays markedly facilitate the collection and computer processing of data (Figures 2D,E). Here, we argue that the two strategies can now be co-applied in zebrafish screening studies, resulting in “*higher-throughput*” screens capable of generating “super-big data” (Figures 2D,E, Glossary). Enhancing the efficiency of zebrafish-based models for innovative CNS drug discovery, this strategy combines the advantages of the extensive approach (utilizing large numbers of drugs and animals tested) with the benefits of using extensive studies (focusing on a large number of phenotypes that help generate mechanistic insights; Figures 2D,E).

In addition to zebrafish's utility for HTS *per se*, this species also enjoys the advantage of having a sophisticated molecular genetic toolset developed for it. For example, traditional N-ethyl-N-nitrosourea (ENU)-induced (Mullins et al., 1994) or viral vector-mediated (Amsterdam et al., 1999) mutagenesis and gene silencing using morpholinos complement other genetic tools recently developed for zebrafish, such as “gene-breaking transposon” (GBT) (Petzold et al., 2009) screens, “clustered regularly interspaced short palindromic repeats” (CRISPR) (Hwang et al., 2013), zinc finger nucleases(Doyon et al., 2008), “transcription activator-like effector nuclease” (TALEN) (Zu et al., 2013) and “targeting induced local lesions in genomes” (TILLING) (Moens et al., 2008). As these genetic tools may manipulate a wide range of zebrafish genes, the ability to apply informatics-driven methods to systematically collect, store and analyze zebrafish pharmacogenetics data further empowers CNS drug discovery. For example, Figure 2E shows how increasing the “dimensionality” of traditional phenotypic screens by including an additional (genetic) dimension becomes a useful strategy of research in this field. Thus, systematic accumulation of pharmacogenetic evidence (with comparative analyses of drug response in wildtype vs. mutant fish) into large online data libraries becomes key for biomolecular data validation and generating novel mechanistic insights into drugs' action (Figure 2E) as part of higher-throughput screening using zebrafish.

## Increasingly sophisticated behavioral screening tools

The higher-throughput screening approach in today's drug discovery also becomes possible due to a combination of rapidly increasing computer processing power with sensitive video-recording tools that generate automated phenotypic data with high spatial and temporal resolution (Figure 2E) (Cachat et al., 2011; Branson, 2014). In addition to testing individual fish with multiple individual endpoints to generate super-big data, simultaneous video-tracking of multiple fish swimming in groups (shoals) (Green et al., 2012; Branson, 2014; Kalueff et al., 2014b) is another strategy to achieve this goal. Moreover, recent advances in automated behavioral recognition (Glossary) further facilitate efficient *in-vivo* drug screens using zebrafish. For example, monitoring several zebrafish body points (e.g., nose, mid-body and tail) enables both automated quantification of fish locomotion and *interpretation* of complex zebrafish behaviors (e.g., chasing, biting, social approach or reduced sociability Kalueff et al., 2013) that parallel human phenotypes (see Stewart et al., 2014a for details).

Finally, as behavioral analyses become more efficient, stimuli that induce fish responses also become better standardized in zebrafish models. For example, currently available automated drug and/or food delivery tools improve zebrafish studies of drugs that affect reward mechanisms. Likewise, zebrafish operant behavior can be examined by using changes in zebrafish body position (e.g., swimming to a specific location) to deliver behavioral stimuli, such as punishment or reward. Exposing fish to predator or conspecific images on a computer screen (Luca and Gerlai, 2012), as well as using a computer-animated robotic “fish” (Butail et al., 2013; Cianca et al., 2013), represent other excellent examples of improved controllability of stimulus presentation in zebrafish models. Because stimulus delivery, behavioral quantification and phenotype recognition/interpretation are now increasingly computerized, zebrafish-based *in-vivo* screening in general is becoming more automated and higher-throughput.

## Concluding remarks

Fully automated robot-based screening platforms for larval and, eventually, adult zebrafish will soon become routine in zebrafish neurophenotyping studies. The HTS systems developed specifically for CNS drugs that affect complex behaviors will increase both the “width” and the “depth” of such screens (Figures 2D,E). Coupled with HTS, the ever increasing sophistication of genetic and behavior-recognition tools transform zebrafish models into higher-throughput *in-vivo* assays, allowing comprehensive coverage of the biological mechanisms of complex brain disorders, and leading to innovative CNS drug discovery.

### Conflict of interest statement

The Review Editor Dr. Caroline Helen Brennan declares that, despite having collaborated with the author Dr. Allan V. Kalueff, the review process was handled objectively. The authors declare that the research was conducted in the absence of any commercial or financial relationships that could be construed as a potential conflict of interest.

## Glossary

***Barcoding*** is a phenotyping approach that expresses complex behavioral changes by splitting them into simple behavioral “barcodes” (Kokel et al., 2010, 2012), to classify psychotropic drugs and determine their mechanisms of action. This approach streamlines and facilitates the analysis of zebrafish phenotypes by providing a concise quantitative and visualized summary of the observed behaviors (Figures 2D,E) (Kokel et al., 2010, 2012). Typically applied to larval fish, this approach has recently been applied to adult zebrafish (Maximino et al., 2014a).

***Behavioral recognition*** complements traditional behavioral quantification methods (e.g., Supplementary Table 1S online) and analyzes the location and temporal dynamics of multiple body parts, in order to interpret complex behaviors. For instance, moving fast nose-to-tail with another fish represents chasing, fast moving head toward another fish is attack, whereas slow moving head to head of another fish can be interpreted as social investigation (Kalueff et al., 2013). Behavioral recognition is markedly enhanced by using computer-based automated videotracking combined with analyses of multiple body parts (Stewart et al., 2014a).

***“Big data”*** represents collected data sets which are difficult to process using traditional data processing approaches due to their size/density (e.g., currently, big data involves millions of terabites of information). In zebrafish screens, big data can be generated using sophisticated automated computer-based video-tracking tools, recording spatial coordinates of zebrafish and their alterations over time.

***Extensive strategy*** in zebrafish drug discovery (Figures 2D,E) employs multiple zebrafish (large animal numbers, N) to rapidly screen many CNS drugs (large D's) and identify novel promising compounds, based on alteration in selected well-validated individual phenotypes (small P's).

***High-throughput screens (HTS)*** in zebrafish involve rapid testing of a very large number of compounds (typically >500–1000 per day) vs. medium- or low-throughput screens. The process often entails a high degree of automation. Traditionally, HTS utilize an extensive strategy (Figure 2D), yet are limited in their ability to thoroughly examine large numbers of endpoints and their associated patterns.

***Intensive strategy*** in zebrafish drug discovery (Figure 2D) focuses on in-depth analyses of multiple phenotypes (large P's) evoked by a small number of experimental manipulations (e.g., drugs, small D's) in a fewer animals (small N's), aiming to increase our mechanistic understanding of various CNS processes and their experimental modulation.

***Neurophenomics*** is an area of neuroscience research (part of phenomics) that examines the interactions between various CNS phenotypes (e.g., zebrafish behaviors) and their response to endogenous (e.g., genetic mutation) or exogenous (e,g., drugs, environmental stressors) factors. A primary aim of zebrafish neurophenomics is to increase the ability to measure and dissect various phenotypes (e.g., by using HTS and test batteries).

***Higher-throughput screening*** in zebrafish neurophenomics combines principles of intensive and extensive screening high-throughput approaches to generate a higher-density data (“super-big data,” Figure 2D). Specifically, the extensive nature of conventional zebrafish HTS is utilized while further assessing in-depth multiple novel endpoints and motor patterning. In general, the higher-throughput strategy “digs deeper” as it uncovers rich phenotypical information hidden in datasets, while still maintaining the rapid and expansive output of HTS.

***Three-dimensional (3D) phenotyping*** is an approach that is based on recording zebrafish locomotor responses, such as 3D swim traces in XYZ coordinates, by several cameras (e.g., two angled cameras as in Figure 1B) (Cachat et al., 2011), enabling in-depth characterization of phenotypic patterns after acute or chronic drug treatment.

***Video-tracking*** is a method of behavioral registration in a single or grouped zebrafish (Figure 1A), using computer-generated 2D (e.g., top- or side-view recording) or 3D phenotyping analyses. Importantly, video-tracking technology is highly automated, and offers the ability to measure a wide array of endpoints not quantifiable through manual observation (Cachat et al., 2011; Branson, 2014).

## References

[B1] Amsterdam A. Burgess S. Golling G. Chen W. Sun Z. Townsend K.. (1999). A large-scale insertional mutagenesis screen in zebrafish. Genes Dev. 13, 2713–2724. 10.1101/gad.13.20.2713 10541557 PMC317115

[B2] Baraban S. C. Taylor M. R. Castro P. A. Baier H. (2005). Pentylenetetrazole induced changes in zebrafish behavior, neural activity and c-fos expression. Neuroscience 131, 759–768. 10.1016/j.neuroscience.2004.11.031 15730879

[B3] Bernier R. Golzio C. Xiong B. Stessman H. A. Coe B. P. Penn O.. (2014). Disruptive CHD8 mutations define a subtype of autism early in development. Cell 158, 263–276. 10.1016/j.cell.2014.06.017 24998929 PMC4136921

[B4] Branson K. (2014). Distinguishing seemingly indistinguishable animals with computer vision. Nat. Methods 11, 721–722. 10.1038/nmeth.3004 24972171

[B5] Bruni G. Lakhani P. Kokel D. (2014). Discovering novel neuroactive drugs through high-throughput behavior-based chemical screening in the zebrafish. Front. Pharmacol. 5:153. 10.3389/fphar.2014.00153 25104936 PMC4109429

[B6] Butail S. Bartolini T. Porfiri M. (2013). Collective response of zebrafish shoals to a free-swimming robotic fish. PLoS ONE 8:e76123. 10.1371/journal.pone.0076123 24146825 PMC3797741

[B7] Cachat J. Kyzar E. J. Collins C. Gaikwad S. Green J. Roth A.. (2013). Unique and potent effects of acute ibogaine on zebrafish: the developing utility of novel aquatic models for hallucinogenic drug research. Behav. Brain Res. 236, 258–269. 10.1016/j.bbr.2012.08.041 22974549

[B8] Cachat J. Stewart A. Grossman L. Gaikwad S. Kadri F. Chung K. M.. (2010). Measuring behavioral and endocrine responses to novelty stress in adult zebrafish. Nat. Protoc. 5, 1786–1799. 10.1038/nprot.2010.140 21030954

[B9] Cachat J. Stewart A. Utterback E. Hart P. Gaikwad S. Wong K.. (2011). Three-dimensional neurophenotyping of adult zebrafish behavior. PLoS ONE 6:e17597. 10.1371/journal.pone.0017597 21408171 PMC3049776

[B10] Cianca V. Bartolini T. Porfiri M. Macri S. (2013). A robotics-based behavioral paradigm to measure anxiety-related responses in zebrafish. PLoS ONE 8:e69661. 10.1371/journal.pone.0069661 23922773 PMC3726767

[B11] Doyon Y. McCammon J. M. Miller J. C. Faraji F. Ngo C. Katibah G. E.. (2008). Heritable targeted gene disruption in zebrafish using designed zinc-finger nucleases. Nat. Biotechnol. 26, 702–708. 10.1038/nbt1409 18500334 PMC2674762

[B12] Duman R. S. Heninger G. R. Nestler E. J. (1994). Molecular psychiatry. Adaptations of receptor-coupled signal transduction pathways underlying stress- and drug-induced neural plasticity. J. Nerv. Ment. Dis. 182, 692–700. 10.1097/00005053-199412000-00003 7989913

[B13] Egan R. J. Bergner C. L. Hart P. C. Cachat J. M. Canavello P. R. Elegante M. F.. (2009). Understanding behavioral and physiological phenotypes of stress and anxiety in zebrafish. Behav. Brain Res. 205, 38–44. 10.1016/j.bbr.2009.06.022 19540270 PMC2922906

[B14] Gerlai R. Lahav M. Guo S. Rosenthal A. (2000). Drinks like a fish: zebra fish (*Danio rerio*) as a behavior genetic model to study alcohol effects. Pharmacol. Biochem. Behav. 67, 773–782. 10.1016/S0091-3057(00)00422-6 11166068

[B15] Green J. Collins C. Kyzar E. J. Pham M. Roth A. Gaikwad S.. (2012). Automated high-throughput neurophenotyping of zebrafish social behavior. J. Neurosci. Methods 210, 266–271. 10.1016/j.jneumeth.2012.07.017 22884772

[B16] Griebel G. Holmes A. (2013). 50 years of hurdles and hope in anxiolytic drug discovery. Nat. Rev. Drug Discov. 12, 667–687. 10.1038/nrd4075 23989795 PMC4176700

[B17] Griffiths B. B. Schoonheim P. J. Ziv L. Voelker L. Baier H. Gahtan E. (2012). A zebrafish model of glucocorticoid resistance shows serotonergic modulation of the stress response. Front. Behav. Neurosci. 6:68. 10.3389/fnbeh.2012.00068 23087630 PMC3468897

[B18] Grossman L. Utterback E. Stewart A. Gaikwad S. Chung K. M. Suciu C.. (2010). Characterization of behavioral and endocrine effects of LSD on zebrafish. Behav. Brain Res. 214, 277–284. 10.1016/j.bbr.2010.05.039 20561961

[B19] Hwang W. Y. Fu Y. Reyon D. Maeder M. L. Tsai S. Q. Sander J. D.. (2013). Efficient genome editing in zebrafish using a CRISPR-Cas system. Nat. Biotechnol. 31, 227–229. 10.1038/nbt.2501 23360964 PMC3686313

[B20] Jin S. Sarkar K. S. Jin Y. N. Liu Y. Kokel D. Van Ham T. J.. (2013). An *in vivo* zebrafish screen identifies organophosphate antidotes with diverse mechanisms of action. J. Biomol. Screen. 18, 108–115. 10.1177/1087057112458153 22960781 PMC4053346

[B21] Kalueff A. V. Echevarria D. J. Stewart A. M. (2014a). Gaining translational momentum: more zebrafish models for neuroscience research. Prog. Neuropsychopharmacol. Biol. Psychiatry 55, 1–6. 10.1016/j.pnpbp.2014.01.022 24593944

[B22] Kalueff A. V. Gebhardt M. Stewart A. M. Cachat J. M. Brimmer M. Chawla J. S.. (2013). Towards a comprehensive catalog of zebrafish behavior 1.0 and beyond. Zebrafish 10, 70–86. 10.1089/zeb.2012.0861 23590400 PMC3629777

[B23] Kalueff A. V. Stewart A. M. Gerlai R. (2014b). Zebrafish as an emerging model for studying complex brain disorders. Trends Pharmacol. Sci. 35, 63–75. 10.1016/j.tips.2013.12.002 24412421 PMC3913794

[B24] Kily L. J. Cowe Y. C. Hussain O. Patel S. McElwaine S. Cotter F. E.. (2008). Gene expression changes in a zebrafish model of drug dependency suggest conservation of neuro-adaptation pathways. J. Exp. Biol. 211, 1623–1634. 10.1242/jeb.014399 18456890

[B25] Kokel D. Bryan J. Laggner C. White R. Cheung C. Y. Mateus R.. (2010). Rapid behavior-based identification of neuroactive small molecules in the zebrafish. Nat. Chem. Biol. 6, 231–237. 10.1038/nchembio.307 20081854 PMC2834185

[B26] Kokel D. Peterson R. T. (2008). Chemobehavioural phenomics and behaviour-based psychiatric drug discovery in the zebrafish. Brief. Funct. Genomic. Proteomic. 7, 483–490. 10.1093/bfgp/eln040 18784194 PMC2722257

[B27] Kokel D. Peterson R. T. (2011). Using the zebrafish photomotor response for psychotropic drug screening. Methods Cell Biol. 105, 517–524. 10.1016/B978-0-12-381320-6.00022-9 21951545 PMC3635141

[B28] Kokel D. Rennekamp A. J. Shah A. H. Liebel U. Peterson R. T. (2012). Behavioral barcoding in the cloud: embracing data-intensive digital phenotyping in neuropharmacology. Trends Biotechnol. 30, 421–425. 10.1016/j.tibtech.2012.05.001 22652049 PMC3401323

[B29] Kyzar E. J. Collins C. Gaikwad S. Green J. Roth A. Monnig L.. (2012). Effects of hallucinogenic agents mescaline and phencyclidine on zebrafish behavior and physiology. Prog. Neuropsychopharmacol. Biol. Psychiatry 37, 194–202. 10.1016/j.pnpbp.2012.01.003 22251567 PMC3294104

[B30] Laggner C. Kokel D. Setola V. Tolia A. Lin H. Irwin J. J.. (2012). Chemical informatics and target identification in a zebrafish phenotypic screen. Nat. Chem. Biol. 8, 144–146. 10.1038/nchembio.732 22179068 PMC3262069

[B31] Lange M. Norton W. Coolen M. Chaminade M. Merker S. Proft F.. (2012a). The ADHD-linked gene Lphn3.1 controls locomotor activity and impulsivity in zebrafish. Mol. Psychiatry 17, 855. 10.1038/mp.2012.119 22918194

[B32] Lange M. Norton W. Coolen M. Chaminade M. Merker S. Proft F.. (2012b). The ADHD-susceptibility gene lphn3.1 modulates dopaminergic neuron formation and locomotor activity during zebrafish development. Mol. Psychiatry 17, 946–954. 10.1038/mp.2012.29 22508465

[B33] Li Q. Lin J. Zhang Y. Liu X. Chen X. Q. Xu M. Q.. (2015). Differential behavioral responses of zebrafish larvae to yohimbine treatment. Psychopharmacology (Berl.) 232, 197–208. 10.1007/s00213-014-3656-5 24958231

[B34] Luca R. M. Gerlai R. (2012). Animated bird silhouette above the tank: acute alcohol diminishes fear responses in zebrafish. Behav. Brain Res. 229, 194–201. 10.1016/j.bbr.2012.01.021 22266470 PMC3293988

[B35] Markou A. Chiamulera C. Geyer M. A. Tricklebank M. Steckler T. (2009). Removing obstacles in neuroscience drug discovery: the future path for animal models. Neuropsychopharmacology 34, 74–89. 10.1038/npp.2008.173 18830240 PMC2651739

[B36] Mathur P. Berberoglu M. A. Guo S. (2011a). Preference for ethanol in zebrafish following a single exposure. Behav. Brain Res. 217, 128–133. 10.1016/j.bbr.2010.10.015 20974186 PMC3466104

[B37] Mathur P. Guo S. (2011). Differences of acute versus chronic ethanol exposure on anxiety-like behavioral responses in zebrafish. Behav. Brain Res. 219, 234–239. 10.1016/j.bbr.2011.01.019 21255611 PMC3062742

[B38] Mathur P. Lau B. Guo S. (2011b). Conditioned place preference behavior in zebrafish. Nat. Protoc. 6, 338–345. 10.1038/nprot.2010.201 21372814 PMC6233885

[B39] Maximino C. Da Silva A. W. Araujo J. Lima M. G. Miranda V. Puty B.. (2014a). Fingerprinting of psychoactive drugs in zebrafish anxiety-like behaviors. PLoS ONE 9:e103943. 10.1371/journal.pone.0103943 25079766 PMC4117595

[B40] Maximino C. Da Silva A. W. Gouveia A. Jr. Herculano A. M. (2011). Pharmacological analysis of zebrafish (*Danio rerio*) scototaxis. Prog. Neuropsychopharmacol. Biol. Psychiatry 35, 624–631. 10.1016/j.pnpbp.2011.01.006 21237231

[B41] Maximino C. De Brito T. M. Colmanetti R. Pontes A. A. De Castro H. M. De Lacerda R. I.. (2010). Parametric analyses of anxiety in zebrafish scototaxis. Behav. Brain Res. 210, 1–7. 10.1016/j.bbr.2010.01.031 20117146

[B42] Maximino C. Lima M. G. Costa C. C. Guedes I. M. Herculano A. M. (2014b). Fluoxetine and WAY 100,635 dissociate increases in scototaxis and analgesia induced by conspecific alarm substance in zebrafish (*Danio rerio* Hamilton 1822). Pharmacol. Biochem. Behav. 124, 425–433. 10.1016/j.pbb.2014.07.003 25019652

[B43] Maximino C. Puty B. Benzecry R. Araujo J. Lima M. G. De Jesus Oliveira Batista E.. (2013). Role of serotonin in zebrafish (*Danio rerio*) anxiety: relationship with serotonin levels and effect of buspirone, WAY 100635, SB 224289, fluoxetine and para-chlorophenylalanine (pCPA) in two behavioral models. Neuropharmacology 71, 83–97. 10.1016/j.neuropharm.2013.03.006 23541719

[B44] Mitchell K. (2011). The miswired brain: making connections from neurodevelopment to psychopathology. BMC Biol. 9:23. 10.1186/1741-7007-9-23 21489316 PMC3076292

[B45] Moens C. B. Donn T. M. Wolf-Saxon E. R. Ma T. P. (2008). Reverse genetics in zebrafish by TILLING. Brief. Funct. Genomic. Proteomic. 7, 454–459. 10.1093/bfgp/eln046 19028802 PMC2899843

[B46] Mullins M. C. Hammerschmidt M. Haffter P. Nüsslein-Volhard C. (1994). Large-scale mutagenesis in the zebrafish: in search of genes controlling development in a vertebrate. Curr. Biol. 4, 189–202. 10.1016/S0960-9822(00)00048-8 7922324

[B47] Neelkantan N. Mikhaylova A. Stewart A. M. Arnold R. Gjeloshi V. Kondaveeti D.. (2013). Perspectives on zebrafish models of hallucinogenic drugs and related psychotropic compounds. ACS Chem. Neurosci. 4, 1137–1150. 10.1021/cn400090q 23883191 PMC3750682

[B48] Nestler E. J. (2013). The origins of molecular psychiatry. J. Mol. Psychiatry 1:3. 10.1186/2049-9256-1-3 25408896 PMC4223876

[B49] Norton W. H. Stumpenhorst K. Faus-Kessler T. Folchert A. Rohner N. Harris M. P.. (2011). Modulation of Fgfr1a signaling in zebrafish reveals a genetic basis for the aggression-boldness syndrome. J. Neurosci. 31, 13796–13807. 10.1523/JNEUROSCI.2892-11.2011 21957242 PMC6633160

[B50] Pan Y. Kaiguo M. Razak Z. Westwood J. T. Gerlai R. (2011). Chronic alcohol exposure induced gene expression changes in the zebrafish brain. Behav. Brain Res. 216, 66–76. 10.1016/j.bbr.2010.07.017 20654657 PMC2975900

[B51] Parker M. O. Brock A. J. Millington M. E. Brennan C. H. (2013). Behavioural phenotyping of casper mutant and 1-pheny-2-thiourea treated adult zebrafish. Zebrafish 10, 466–471. 10.1089/zeb.2013.0878 23869690 PMC4167592

[B52] Petzold A. M. Balciunas D. Sivasubbu S. Clark K. J. Bedell V. M. Westcot S. E.. (2009). Nicotine response genetics in the zebrafish. Proc. Natl. Acad. Sci. 106, 18662–18667. 10.1073/pnas.0908247106 19858493 PMC2767365

[B53] Rihel J. Prober D. A. Arvanites A. Lam K. Zimmerman S. Jang S.. (2010). Zebrafish behavioral profiling links drugs to biological targets and rest/wake regulation. Science 327, 348–351. 10.1126/science.1183090 20075256 PMC2830481

[B54] Rosemberg D. B. Rico E. P. Mussulini B. H. Piato A. L. Calcagnotto M. E. Bonan C. D.. (2011). Differences in spatio-temporal behavior of zebrafish in the open tank paradigm after a short-period confinement into dark and bright environments. PLoS ONE 6:e19397. 10.1371/journal.pone.0019397 21559304 PMC3085514

[B55] Soleymani A. Cachat J. M. Robinson K. Dodge S. Kalueff A. Wurbel R. (2014). Integrating cross-scale analysis in the spatial and temporal domains for classification of behavioral movement. J. Spatial Inform. Sci. 8, 1–25 10.5311/JOSIS.2014.8.162

[B56] Stewart A. M. Braubach O. Spitsbergen J. Gerlai R. Kalueff A. V. (2014a). Zebrafish models for translational neuroscience research: from tank to bedside. Trends Neurosci. 37, 264–278. 10.1016/j.tins.2014.02.011 24726051 PMC4039217

[B57] Stewart A. M. Cachat J. Gaikwad S. Robinson K. S. Gebhardt M. Kalueff A. V. (2013). Perspectives on experimental models of serotonin syndrome in zebrafish. Neurochem. Int. 62, 893–902. 10.1016/j.neuint.2013.02.018 23485557

[B58] Stewart A. M. Grossman L. Nguyen M. Maximino C. Rosemberg D. B. Echevarria D.. (2014b). Aquatic toxicology of fluoxetine:understanding the knowns and the unknowns. Aquat. Toxicol. 156, 269–273. 10.1016/j.aquatox.2014.08.014 25245382

[B59] Stewart A. M. Kalueff A. V. (2013). Controlled substances and innovation of biomedicine: a preclinical perspective. Nat. Rev. Neurosci. 14, 877. 10.1038/nrn3530-c1 24149185

[B60] Stewart A. M. Kalueff A. V. (2014). Anxiolytic drug discovery: what are the novel approaches and how can we improve them? Expert Opin. Drug Discov. 9, 15–26. 10.1517/17460441.2014.857309 24206163

[B61] WHO. (2008). The Global Burden of Disease, 2004 Update. Geneva: WHO.

[B62] Wong K. Stewart A. Gilder T. Wu N. Frank K. Gaikwad S.. (2010). Modeling seizure-related behavioral and endocrine phenotypes in adult zebrafish. Brain Res. 1348, 209–215. 10.1016/j.brainres.2010.06.012 20547142

[B63] Ziv L. Muto A. Schoonheim P. J. Meijsing S. H. Strasser D. Ingraham H. A.. (2013). An affective disorder in zebrafish with mutation of the glucocorticoid receptor. Mol. Psychiatry 18, 681–691. 10.1038/mp.2012.64 22641177 PMC4065652

[B64] Zu Y. Tong X. Wang Z. Liu D. Pan R. Li Z.. (2013). TALEN-mediated precise genome modification by homologous recombination in zebrafish. Nat. Methods 10, 329–331. 10.1038/nmeth.2374 23435258

